# Supplementary material for: Toxic Effects on Thyroid Gland of Male Adult Lizards (Podarcis Siculus) in Contact with PolyChlorinated Biphenyls (PCBs)-Contaminated Soil
Source: Int J Mol Sci. 2022 Apr 26;23(9):4790. doi: 10.3390/ijms23094790 (PMC9102276; doi:10.3390/ijms23094790)
Supplement: Supplementary file 1 [file ijms-23-04790-s001.zip › ijms-1671494-supplementary.pdf]

**TREATMENTS**	**T_3/T_4**
Control in nonpolluted terraria	0.696
Control with oil	0.560
GROUP A	0.581[a]
GROUP B	0.655[b]
GROUP C	0.808[d]
GROUP D	0.730[c]

Table S1- The T_3 to T_4 ratio after exposure to 2.50 (Group A), 4.50 (Group B), 7.50 (Group C) mg/kg PCBs and after treatment with a single dose of Aroclor 1242, 1254 and 1260, cyto-chrome P4501A and P4502B (Group D) in the comparison with the different controls. The letters indicate significant differences be-tween the different groups from each period ($p < 0.05$).

Low chlorinated PCBs
PCB 28
PCB 30
PCB 31
PCB 52
PCB77
PCB81
PCB96
PCB99
Medium chlorinated PCBs
PCB 101
PCB105
PCB110
PCB114
PCB118
PCB123
PCB126
PCB128
PCB138
PCB146
PCB149
PCB151
PCB 156
PCB157
PCB167
PCB 169
High chlorinated PCBs
PCB170
PCB 177
PCB 180
PCB 183
PCB187
PCB 189

Table S2. The single 31 congeners present in three group of contaminated soils.
